# Genomic Landscapes of Endometrioid and Mucinous Ovarian Cancers and Morphologically Similar Tumor Types

**DOI:** 10.1158/2767-9764.CRC-25-0147

**Published:** 2025-11-05

**Authors:** Dorothy Hallberg, Alice C. Eastman, Shashikant Koul, Daniel C. Bruhm, Eniko Papp, Simon Davenport, Vilmos Adleff, Leonardo Ferreira, Noushin Niknafs, Jamie E. Medina, Stephen Cristiano, Carolyn Hruban, Jacob Fiksel, Kaui P. Lebarbenchon, Luis Aparicio, Nicholas A. Vulpescu, Kuan-Ting Kuo, Nita Ahuja, Ronny Drapkin, Euihye Jung, Sarah H. Kim, Mark A. Eckert, Ernst Lengyel, Kentaro Nakayama, Ayse Ayhan, Ie-Ming Shih, Tian-Li Wang, Ofer Lavie, Gad Rennert, Hariharan Easwaran, Stephen B. Baylin, Michael F. Press, Victor E. Velculescu, Robert B. Scharpf

**Affiliations:** 1 The Sidney Kimmel Comprehensive Cancer Center, Johns Hopkins University School of Medicine, Baltimore, Maryland.; 2 Department of Pathology, Norris Comprehensive Cancer Center, University of Southern California, Los Angeles, California.; 3 Department of Pathology, National Taiwan University Hospital, Taipei, Taiwan.; 4 Department of Pathology and Surgery, Yale University School of Medicine, New Haven, Connecticut.; 5 Division of Gynecologic Oncology, Department of Obstetrics and Gynecology, Penn Ovarian Cancer Research Center, Hospital of the University of Pennsylvania and Perelman School of Medicine, University of Pennsylvania, Philadelphia, Pennsylvania.; 6 Department of Obstetrics and Gynecology and Section of Gynecologic Oncology, The University of Chicago, Chicago, Illinois.; 7 Department of Gynecology and Obstetrics, Nagoya City University East Medical Center, Nagoya, Japan.; 8 Department of Pathology, Seirei Mikatahara Hospital, Hamamatsu, Japan.; 9 Department of Tumor Pathology, Hamamatsu University School of Medicine, Hamamatsu, Japan.; 10 Department of Molecular Pathology, Hiroshima University School of Medicine, Hiroshima, Japan.; 11 Division of Obstetrics and Gynecology, Carmel Medical Center, Haifa, Israel.; 12 B. Rappaport Faculty of Medicine, Technion-Israel Institute of Technology and The Association for Promotion of Research in Precision Medicine, Haifa, Israel.; 13 Department of Biostatistics, Johns Hopkins Bloomberg School of Public Health, Baltimore, Maryland.

## Abstract

Endometrioid and mucinous ovarian carcinomas represent nearly a fifth of ovarian cancers, but their molecular characteristics and pathologic origins are poorly understood. To identify the genomic and epigenomic alterations characteristic of these ovarian cancer subtypes and evaluate links to morphologically similar tumors from other sites, we performed a combination of sequence, copy number, mutation signature, and rearrangement analyses from tumor samples and matched normal tissues of 133 patients, as well as methylation analyses of these tumors and tissues of 150 patients from The Cancer Genome Atlas. Genomic analyses included samples from patients with ovarian endometrioid (*n* = 44), ovarian mucinous (*n* = 43), uterine endometrioid (*n* = 15), and gastrointestinal mucinous carcinomas (*n* = 31), including mucinous carcinomas of the stomach, colon, and pancreas. In addition to identifying genes previously known to be involved in these tumors, we identified alterations in *RAD51C*, *NOTCH4*, *SMARCA1/4*, and *JAK1* in ovarian endometrioid, *ESR1* in uterine endometrioid, and *SMARCA4* in ovarian mucinous carcinomas. Whole-genome sequencing revealed rearrangements involving *PTEN*, *NF1*, and *NF2* in ovarian endometrioid carcinomas and *NF1* and *MED1* in ovarian mucinous carcinomas. The number of alterations, affected genes, and genome-wide methylation profiles were not distinguishable between ovarian and uterine endometrioid carcinomas, supporting the hypothesis that these tumors share a tissue of origin. In contrast, mutation and methylation patterns in ovarian mucinous carcinomas were different from gastrointestinal mucinous carcinomas. These analyses provide insights into the genomic landscapes and origins of mucinous and endometrioid ovarian carcinomas, providing new avenues for early clinical intervention and management of patients with these cancers.

**Significance:**

Integrative multi-omic analyses support a common tissue of origin between ovarian endometrioid and uterine endometrioid carcinomas but not between ovarian mucinous and gastric or pancreatic mucinous carcinomas.

## Introduction

Ovarian cancer is the leading cause of gynecologic cancer death and is the seventh most common cancer in women worldwide. Epithelial ovarian cancer comprises about 90% of all ovarian malignancies and includes high-grade serous, low-grade serous, mucinous, endometrioid, and clear cell ovarian carcinomas. High-grade serous ovarian carcinomas (HGSOC) represent more than half of all epithelial ovarian cancers and is the most widely studied epithelial ovarian tumor, whereas endometrioid and mucinous carcinomas account for 10% and 6%, respectively (1). Through extensive whole-genome sequencing (WGS), whole-exome sequencing (WES), and methylation analyses of HGSOC, a variety of genes have been identified as likely drivers in these tumors (2), and both clinical and evolutionary analyses have implicated the fallopian tube as the tissue of origin of HGSOC (3–11). Apart from alterations in a few well-known drivers, including *KRAS* and *HER2* amplification, in ovarian serous and mucinous carcinomas and mutations in *ARID1A*, *PIK3CA*, and *PTEN* genes as well as mismatch repair (MMR) deficiency in ovarian endometrioid carcinomas, comparatively little is known about molecular alterations in these tumors (12). Comprehensive genomic analyses of these tumor subtypes could delineate additional alterations and pathways frequently altered in these cancers and shed light on their likely tissue of origin.

Uterine endometrioid adenocarcinomas account for nearly 85% of all epithelial uterine carcinomas, and accumulating evidence suggests a shared molecular etiology between these cancers and ovarian endometrioid adenocarcinomas (13). Synchronous endometrioid ovarian and uterine carcinomas can arise from independent primary tumors or as metastases from a single primary tumor (14, 15), suggesting that these tumors may share the same cell of origin (16, 17). Women with synchronous ovarian and uterine cancers have improved survival compared with women with a single cancer, in part due to early diagnosis from the symptoms of vaginal bleeding associated with uterine carcinomas (18). Understanding the origin of these cancers could lead to more accurate prognoses of survival or more aggressive early intervention. Whereas WGS, WES, and targeted next-generation sequencing analyses have been performed on ovarian endometrioid carcinomas (19–21), and whole-exome studies have been performed on uterine endometrioid carcinomas (22), the extent to which these cancers share similar alterations and a potentially common tissue of origin has not been systematically evaluated.

**Figure 1.**
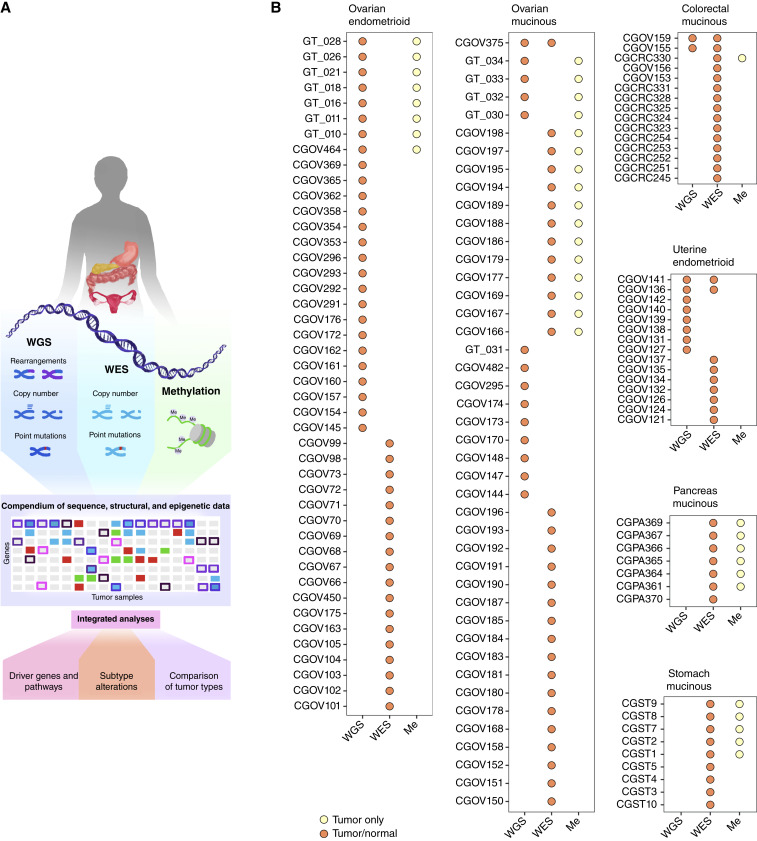
Overview of genomic and epigenomic analyses. **A,** We performed genomic analyses of tumor and matched white blood cell samples from patients with ovarian mucinous, ovarian endometrioid, uterine endometrioid, and GI mucinous carcinomas to identify sequence, structural, and epigenetic changes using WGS, WES, and Infinium MethylationEPIC arrays. We integrated these data to characterize and evaluate differences between the commonly altered pathways and cancer driver genes in these histologic subtypes. **B,** Overview of the availability of genomic, exomic, and methylation (Me) for the ovarian, uterine, and GI carcinomas.

Distinguishing between primary mucinous ovarian cancer and mucinous adenocarcinoma metastases from other sites, including gastrointestinal (GI) mucinous tumors, may be challenging. Some histologic features can favor primary diagnosis, but there are several discordant and overlapping features that make a definitive diagnosis difficult (23). Molecular analyses for mucinous ovarian and GI cancers, including pancreatic, stomach and colorectal cancers, have largely been limited to targeted sequencing (24–28), gene expression (29), or immunohistochemistry (IHC; ref. 30), though recently work has begun on genomic analyses of these malignancies (27, 31). No studies to date have compared the epigenomes of mucinous carcinomas from the ovary with those of other sites. Such analyses could establish the tissue of origin and lead to better risk stratification and treatment for patients with these cancers. In this study, we characterize the molecular landscapes of endometrioid and mucinous adenocarcinomas, comparing genomes and epigenomes in both the cancer and adjacent normal tissues (Fig. 1A). Our analyses indicate the major pathways affected by these cancers and identify molecular alterations that may improve our understanding of their tissue of origin.

## Materials and Methods

### Specimens obtained for sequencing analysis

The diagnosis of primary ovarian carcinoma was made at the at the referral institution and required not only the presence of an adnexal mass but also no evidence of malignancy in mucosal surfaces of the digestive tract or uterus. The ovarian cancer subtype was diagnosed by board-certified pathologists at the referral institution and subsequently confirmed by a central laboratory board-certified pathologist. Matched tumor and normal tissue were collected from patients at the time of resection (Supplementary Table S1). The matched normal tissue was obtained from white blood cells isolated from peripheral blood. When peripheral blood was not available, connective tissue adjacent to the tumor that contained no tumor by histopathologic examination was used as the source of normal DNA. The percent viable tumor cell content was determined by histopathologic assessment. All samples were obtained under Institutional Review Board–approved protocols with informed consent for research use at participating institutions.

### Sample preparation and next-generation sequencing

DNA was extracted from patient whole blood using a QIAamp DNA Blood Mini QIAcube Kit (Qiagen). Microdissection was performed using a scalpel and dissecting microscope with comparison of unstained sections to be dissected to a serial section that was stained with hematoxylin and eosin. Genomic DNA from formalin-fixed, paraffin-embedded (FFPE) blocks was extracted from the microdissected tissues using the QIAamp DNA FFPE Tissue Kit (Qiagen). In brief, the samples were incubated in proteinase K for 16 hours before DNA extraction. The digested mixture was transferred to a microtube for DNA fragmentation using the truXTRACTM FFPE DNA Kit with 10 minutes shearing time as per the manufacturer’s instructions (Covaris). Following fragmentation, the sample was further digested for 24 hours followed by 1-hour incubation at 80°C. DNA purification was performed using the QIAamp DNA FFPE Tissue Kit following the manufacturer’s instructions (Qiagen). Fragmented genomic DNA from tumor and normal samples were used for Illumina TruSeq library construction (Illumina) according to the manufacturer’s instructions or as previously described (32). Exonic or targeted regions were captured in solution using the Agilent SureSelect v.4 Kit or a custom targeted panel according to the manufacturer’s instructions (Agilent). Paired-end sequencing, 100 bases from each end of the fragments for exome libraries and 150 bases from each end of the fragment for targeted libraries, was performed using Illumina HiSeq 2000/2500 and Illumina MiSeq instrumentation (Illumina). WGS for tumor and matched normal samples was performed at an average depth of 60× and 30×, respectively, whereas WES was performed at an average depth of 700× and 400×, respectively (Supplementary Table S2).

### Genotype concordance of matched samples

Where multiple tissue samples were available from a single patient, we assessed the concordance of genotypes at SNPs genome-wide. The dbSNP build 130 (reference genome hg18) was downloaded from the UCSC Genome Browser (RRID: SCR_005780). Only positions of single-base substitutions were retained from which 10,000 positions were randomly sampled. Rsamtools (v2.16.0) was used to generate pileup statistics for each BAM file at the filtered dbSNP positions. The pileup was evaluated at positions with mapping and base quality scores greater than 30. Positions were considered evaluable only if there was at least 20 spanning reads and 95% of reads supported the same base. For every pair of BAM files, the proportion of concordant genotypes was calculated as the ratio of matched bases to the total number of evaluated bases. Using the distribution of concordance among randomly sampled pairs, we obtained a null distribution of concordance for unrelated individuals. A sample pair was considered genotype-matched if the concordance was an outlier relative to the null distribution (i.e., the concordance exceeded the third quartile plus 1.5 times the IQR of the null). Of 315 samples, 297 samples had genotype concordance that matched the reported tumor–normal pair. When genotype matching differed from available annotation, our analyses used the genotype-matched pairing. We excluded samples from further analyses when no genotype-matched sample was identified or when the annotated tumor types differed between the genotype-matched samples (*n* = 27).

### Mutation analyses

Prior to mutation calling, primary processing of sequence data for both tumor and normal samples were performed using Illumina CASAVA software (v1.8), including masking of adapter sequences. Sequence reads were aligned against the human reference genome version hg18 using ELAND. Candidate somatic mutations, consisting of point mutations, insertions, and deletions, were identified using VariantDx and Strelka as previously described (33, 34). For samples analyzed using targeted sequencing, we identified candidate mutations that were altered in >10% of distinct reads. For samples analyzed using WES, we additionally required ≥5 altered reads in at least one sample. Each candidate mutation was confirmed using BLAT (RRID: SCR_011919) and examined by visual inspection. For each mutation, 101 bases including 50 bases 5′ and 3′ flanking the mutated base was used as query sequence. Candidate mutations were removed if the analyzed region resulted in more than one BLAT hit with 90% identity or a score of at least 70. Mutation signature analyses were performed using the deconstructSigs package in R with default parameters (35).

To model the prevalence of mutations that commonly arise in ovarian endometrioid and uterine endometrioid carcinomas, we identified the set of genes with a combined mutation frequency of at least 10% in these tumors. For a given gene, we denote the number of samples with a mutation in tumor subtype j by y_j_ and x, a dummy variable for uterine endometrioid cancer (0 if ovarian endometrioid and 1 if uterine endometrioid). We modeled the observed mutation frequency as

$$y_{j}\;∼\;\text{Binomial}\text{(}\theta_{j},\;n_{j}\text{)}$$

$$\text{logit}\left(\theta_{j}\right)\;=\;\alpha_{0}+\;\alpha_{1}\;x_{j}$$

$$\alpha_{0}\;∼\;\text{Normal}\left(0,\;10\right)$$

$$\;\alpha_{1}∼\;\text{Double}\;e\text{xponential}\left(0,\;1\right)$$

The prevalence of mutations was obtained from the two available log odds from this model, and the difference in mutation prevalence was obtained from the log OR (LOR) given by α_1_. Ninety percent posterior credible intervals for these parameters were obtained from the 5th and 95th percentiles of these posteriors. Computation was performed using stan and rstan. The same approach was used to model the prevalence of mutations in ovarian mucinous and GI mucinous adenocarcinoma types.

To compute the probability of observing n or more statistically significant differences (α = 0.1) in allele frequencies out of P commonly altered genes under the null that the population allele frequencies are the same for these tumor types, we simulated a 1,000 × P matrix of uniform(0, 1) random deviates. We binarized this matrix such that random deviates <0.1 were assigned the value 1 and zero otherwise. The row-wise sums provide a null distribution for the number of statistically significant genes. A *P* value was obtained by calculating the fraction of observations from the null distribution that were at least as large as the observed number of differences.

### Structural variant analyses

We used FACETS to estimate tumor purity and ploidy (36). Tumor samples for which purity could not be estimated by facets (*n* = 18) or tumors with purity estimates at or below 0.20 (*n* = 11) were excluded from downstream analyses. GC-adjusted log_2_ ratios (log R) were obtained as previously described in both tumor and normal samples (37). To remove technical biases and germline alterations, the log Rs from matched normal samples were subtracted from the corresponding log Rs in the tumor samples. The R package DNAcopy (v1.74.1, RRID: SCR_012560) was used to perform circular binary segmentation on the corrected log Rs (38).

To obtain integer copy number estimates, we modeled the log Rs for a segment i from a sample as a mixture of normal and cancer cells given by the relationship

$$R_{i}=\;\log_{2}\left(\frac{C_{\text{Ti}\;}\times \;p\;+{\;C}_{\text{Ni}}\;\times \;\left(1‐p\right)}{P_{T}\;\times \;p\;+\;P_{N}\;\times \;\left(1‐p\right)}\right)$$

In which p is the tumor purity, C_Ti_ is the number of copies of segment i in the tumor cells, C_Ni_ is the number of copies of segment i in the normal cells, P_T_ is the average ploidy of the tumor cells, and P_N_ is the average ploidy of the normal cells. Log Rs were converted to integer copy numbers for each genomic segment by solving for C_T_ and rounding to the nearest integer. Focal deletions were defined as segments less than 3 Mb in which the integer copy number was either 0 or 1. If two or more improper read pairs supported the deletion, we referred to the segment as hemizygous^+^ or homozygous^+^, with ^+^ denoting the identification of multiple improper read pairs that have alignments consistent with the deletion call. Amplicons were seeded with high-copy focal amplifications of greater than 2.5 times the average ploidy of the sample. Low-copy gains, defined as segments with copy number 1.5 times the average ploidy of the sample, were linked to the seed amplicon if at least two read pairs bridged the low-copy segment to the amplicon.

Somatic copy-neutral intra- and inter-chromosomal translocations and inversions were identified using Trellis as previously described for each tumor sample and its matched normal sample (37). At least two improper read pairs and at least one split read were required to identify rearrangements. Rearrangements in tumor samples that overlapped any rearrangement identified in their matched normal samples were excluded. In-frame gene fusions were identified as previously described (37). Fusions in which the left and right fusion partners were in the same gene and fusions in which none of the exons in the three prime genes were predicted to be in the fusion protein were excluded.

### Methylation analyses

We used the Infinium MethylationEPIC arrays with probes for more than 850,000 CpG sites for genome-wide methylation analysis. Raw IDAT files from the Infinium MethylationEPIC array were preprocessed and normalized using the funnorm function in the R package minfi (39). Probes on chromosomes X or Y, probes with detection *P* value greater than 0.5, or probes overlapping a SNP with dbSNP minor allele frequency greater than 10% were excluded. The proportion of methylated CpG sites was calculated as the fraction of CpG probes with β > 0.3. To evaluate the methylation profiles of these samples in the context of tumor and normal samples from The Cancer Genome Atlas (TCGA), we analyzed 77 colorectal mucinous, 23 stomach mucinous, and 46 uterine endometrial samples obtained from four TCGA studies that were processed on the 450k Infinium Methylation array (Supplementary Table S3). Using 735,052 probes in common with the EPIC array, we identified the 1,000 most variable probes and performed a principal component analysis on the TCGA samples. The five principal components with largest eigenvalues were retained. A linear discriminant (LD) analysis was fit with tissue type as the response (colorectal mucinous, stomach mucinous, or uterine endometrioid) and five principal components as independent variables using the TCGA samples. The tumor samples sequenced in this study were first projected onto the principal components and then onto the LD axes.

### Survival analyses

Time-to-event analyses of overall survival were performed using Kaplan–Meier, and log-rank tests were used to compare differences between Kaplan–Meier survival curves. A pathway was considered altered if a somatic mutation or structural variant (copy number alteration or rearrangement) was identified in any gene belonging to the pathway.

## Results

### Overall approach

A total of 133 ovarian endometrioid, uterine endometrioid, ovarian mucinous, and GI mucinous carcinomas and matching normal tissues were collected from patients of seven institutions in the United States, Israel, Taiwan, and Japan (Fig. 1B; Supplementary Table S1). More than 79% (105 of 133) ovarian and GI mucinous tumor samples had tumor cellularity exceeding 20%, and samples with low cellularity (≤20%) were excluded from downstream analyses (*n* = 28). We performed genomic analyses of these matched tumor and normal tissue samples to identify sequence and structural changes and compared tumor and TCGA methylation data for epigenetic changes (Fig. 1). All samples were analyzed by WES analyses, or, for the subset of cases in which frozen tumors were available, we performed WGS. Overall, we analyzed 73 tumors using WES and 43 tumors using WGS. The median coverage for WES and WGS samples was 535× and 43×, respectively (Supplementary Table S2). We assessed substitutions, small insertions and deletions, as well as copy number changes in samples with WES, whereas samples with WGS were evaluated for these changes as well as for rearrangements, linked amplicons, and gene fusions. Copy number analyses identifying both focal amplifications, deletions, as well as chromosomal gains and losses were performed for WES using FACETS and for WGS using our recently developed Trellis (RRID: SCR_013819) approach (37, 36). We also used Trellis to evaluate linked amplicons that may be derived from an amplification of a single target gene that is connected to multiple different regions of the genome. Finally, we performed methylation analyses using microarrays containing probes for 850,000 CpG sites on 46 samples, including ovarian endometrioid, ovarian mucinous, and colorectal mucinous carcinomas that had remaining genomic DNA, and an additional 22 stomach mucinous and pancreatic mucinous samples.

### Ovarian endometrioid and uterine endometrioid carcinomas

We used a highly sensitive mutation analysis to identify somatic alterations in ovarian endometrioid (*n* = 40) and uterine endometrioid (*n* = 11) cancers (Supplementary Tables S4 and S5). The median number of somatic alterations was 54 for both ovarian endometrioid and uterine endometrioid cancers and did not seem to be associated with tumor purity (Spearman correlation = −0.06, Supplementary Fig. S1; Supplementary Tables S2, S4, and S5). Consistent with previous analyses, we observed a bimodal distribution of the number of mutations for uterine endometrioid carcinomas, with nearly half of the 11 tumors analyzed having more than 500 mutations per exome, likely the result of a hypermutator status (Supplementary Fig. S1; ref. 22). As in previous studies, uterine endometrioid carcinomas were more likely to be hypermutated (4 of 11 patients) compared with ovarian endometrioid carcinomas (3 of 40 patients).

**Figure 2.**
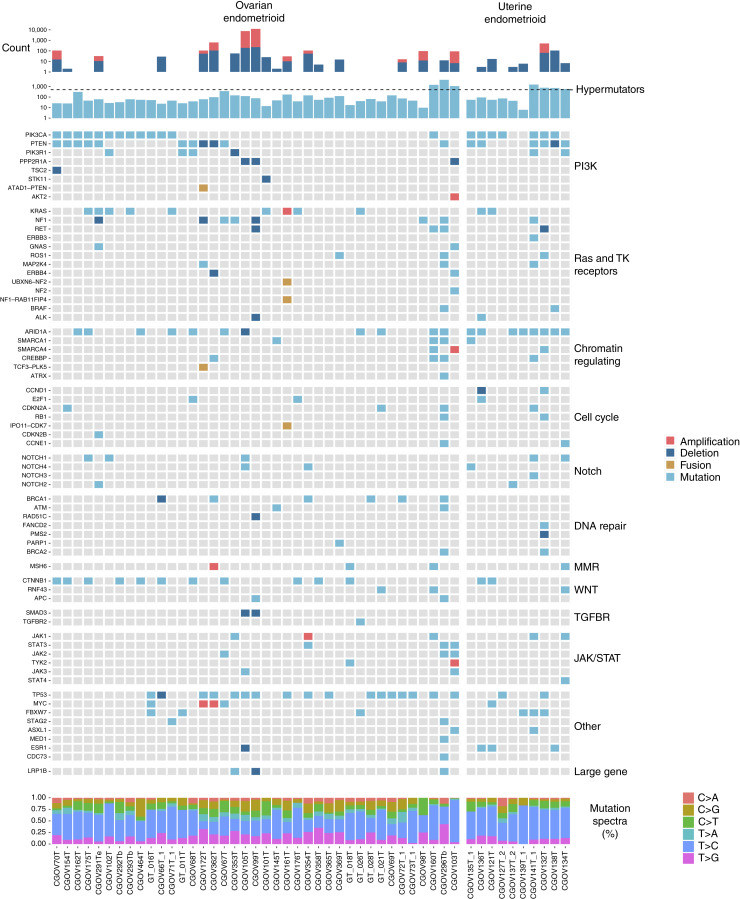
Integrated genomic analyses of ovarian and uterine endometrioid adenocarcinomas. Integrated analyses of somatic point mutations, structural variants, including linked amplicons, deletions, and rearrangements, organized by pathways. The top of the figure shows the number of each type of alteration, the middle portion reflects the alterations present in each sample for each gene and pathway, and the bottom shows the mutation spectra.

Evaluation of the altered genes in ovarian endometrioid and uterine endometrioid carcinomas without a hypermutation phenotype revealed similar affected pathways (Fig. 2). Mutations involving chromatin-regulating genes and PI3K and RAS pathways were common in both tumor types. The proportion of patients with alterations in *PTEN*, *PIK3CA*, *KRAS*, *PIK3R1*, *NF1*, or *ARID1A* genes was 56.1% for ovarian endometrioid and 76.9% for uterine endometrioid, consistent with previous studies (40, 41, 21). As previously reported (42), we observed mutations in *TP53* and *CTNNB1* in 27 patients with ovarian endometrioid carcinomas (68% of patients) and four patients with uterine endometrioid carcinomas (36% of patients). We identified several mutations in driver genes among patients with ovarian endometrioid carcinomas that have not been previously reported, including the homologous recombination *RAD51C* gene, Notch signaling genes (*NOTCH1–4*), chromatin remodeling genes *SMARCA1/4*, transcription factor *E2F1*, receptor tyrosine kinase *RET*, and *ABL1* kinase.

Interestingly, we found that two of the 11 patients with uterine endometrioid carcinomas had alterations in *ESR1*, including two hotspot mutations at Tyr537 (Y537C and Y537N) and an alteration at Leu536 (L536R; Supplementary Fig. S2). The Tyr537 hotspot constitutively activates the estrogen receptor independent of estrogen and has been reported in patients with breast cancer that have become resistant to tamoxifen or aromatase inhibitor therapy (43, 44). Although the consequence of the Leu536 amino acid substitution is not well understood, its adjacency to the Tyr537 hotspot suggests that a similar functional role is likely. Though neither woman with an *ESR1* hotspot mutation had been treated with antiestrogen therapy, both women were above the average age of menopause and would likely have had low levels of estrogen.

To understand the role of mutations in these ovarian cancer subtypes in the context of mutational signatures (45), we evaluated the types of mutations and linked these to mutational processes that include exogenous or endogenous mutagen exposure, enzymatic modification of DNA, and defective DNA repair (Supplementary Fig. S3A). As expected, we found that the age-related mutation signature, signature 1, characterized by a predominance of C>T transitions was present in all samples. Such C>T transitions are believed to be caused by spontaneous deamination of 5-methylcytosine. In patients with hypermutator defects, we identified signatures 6 and 15 known to be associated with DNA MMR deficiency and microsatellite instability (45).

**Figure 3.**
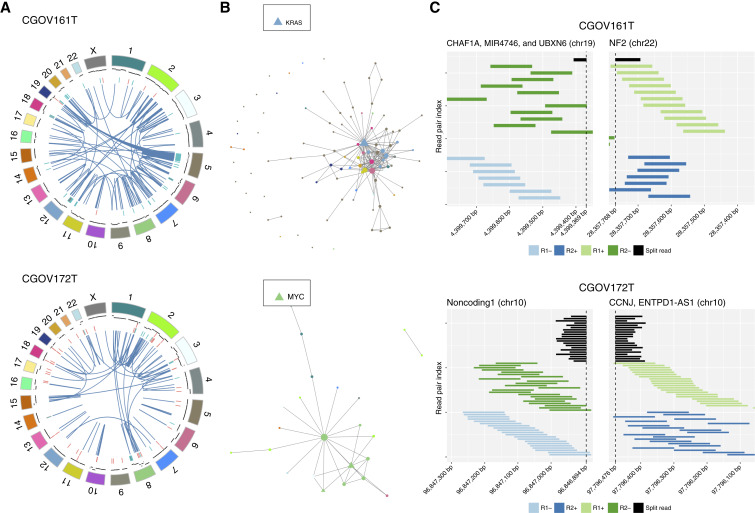
WGS analyses of linked amplicons and rearrangements. **A,** Circos plots for linked amplicons for individual tumor samples with >100 structural variants. Circos plots depict copy number alterations as well as intra- and inter-chromosomal rearrangements. Segmented normalized estimates of read depth were used to identify candidate focal amplifications (teal) and focal deletions (red). Rearranged read pairs and split reads were used to identify inter- and intra-chromosomal rearrangements (blue). **B,** For linked amplicon graphs, nodes represent genes, node size represents the number of times the segment is represented in the genome, color indicates the chromosome location of the amplified segment, and edges denote segments that are connected in the rearranged cancer genome. Known driver genes indicated by triangles are often hubs in these networks, suggesting that amplification of these genes occurs early and can be connected to many of the other amplicons. **C,** Reads aligned to the fusion junction of two representative rearrangements. Split reads that span the fusion junction are shown in black, whereas read pairs that reside on either side of the junction are shown in green and blue, providing additional evidence for specific rearrangements.

In addition to mutations, we evaluated both WES and WGS data for focal amplifications. For WGS data, we examined the read-pair information to pinpoint the presence of amplicons that may be linked genomically using the Trellis approach (Fig. 3A; Supplementary Figs. S4–S7; Supplementary Table S6; ref. 37). The multiple genomic insertion sites as a result of *MYC* and *KRAS* amplification in tumors from patients CGOV161 and CGOV172, respectively, could be connected to other amplicons (Fig. 3B), likely indicating that these genes and the neighboring genomic region at an insertion site were duplicated in successive cell divisions (46). *KRAS* has been shown to be amplified in ovarian endometrioid carcinoma and HGSOC and has been associated with an aggressive phenotype of metastatic uterine endometrioid carcinoma (47, 48), whereas *MYC* amplifications have been observed in a variety of ovarian cancers, including endometrioid carcinomas (49).

We evaluated both WES and WGS data for homozygous deletions and identified losses in multiple genes known to be altered in ovarian endometrioid carcinomas, including *PTEN*, *NF1*, *ARID1A*, *BRCA1*, and others. Additionally, we identified deletions in genes *TSC2* and *STK11* that have not been previously reported in endometrioid ovarian carcinoma (Supplementary Tables S7 and S8). *TSC2* deletions have been observed in HGSOC, and mutations in this gene have been associated with increased sensitivity to rapamycin-induced apoptosis, making *TSC2* a potentially targetable biomarker (50, 51). Germline *STK11* inactivating mutations have been associated with Peutz–Jeghers syndrome (52), and somatic changes in this gene have been reported in a variety of cancers, including non–small cell lung cancer, cervical cancer, colorectal cancer, melanoma, and pancreatic cancer with predominantly truncating mutations but have not yet been reported in endometrioid ovarian carcinoma.

For samples with available WGS, we also used Trellis to identify rearrangements and fusions (Supplementary Fig. S4; Supplementary Table S9). These analyses revealed several novel rearrangements that were predicted to result in in-frame protein fusions in ovarian endometrioid carcinomas, including *ATAD1*–*PTEN*, the neurofibromin genes *NF1* and *NF2*, as well as cyclin-dependent and polo-like kinase (PLK) genes *CDK7* and *PLK5* (Fig. 3C). The *ATAD1*–*PTEN* rearrangement was linked to the homozygous deletion of a portion of *PTEN*. Whereas *PTEN* loss in the ovarian surface epithelium has been shown to induce papillary serous ovarian cancer, rearrangements of *PTEN* associated with deletions have not been previously reported in ovarian endometrioid tumors (50). The neurofibromin gene fusions *NF1*–*RAB11FIP4* and *UBXN6*–*NF2* were identified in patient CGOV161T with ovarian endometrioid carcinoma. Rearrangements involving *NF1* and *NF2* have been reported in other cancers and have been associated with therapeutic resistance in HGSOC (53, 37) but have not been previously identified in ovarian endometrioid carcinoma. Fusions of kinase genes *IPO11*–*CDK7* and *PLK5*–*TCF3* were detected in two different tumors. *CDK7* is involved in cell-cycle progression, and *CDK7* inhibitors have been shown to exert broad cytotoxicity against ovarian tumors (54, 55). The *IPO11* gene is involved in nuclear protein import and is expressed in both ovarian and endometrial cells. The fusion of *IPO11*–*CDK7* may lead to constitutive activation of *CDK7*, promoting cell-cycle progression and tumor growth. The *PLK5* gene has been reported to be altered in glioblastoma and, along with other members of the PLK family, plays a role in cell division and centriole duplication (56, 57). As recent studies suggest that aberrant expression of PLKs affect cell division and influence genomic instability (33, 58), PLKs may provide a useful target for therapeutic interventions (59).

### Ovarian mucinous and GI mucinous cancers

**Figure 4.**
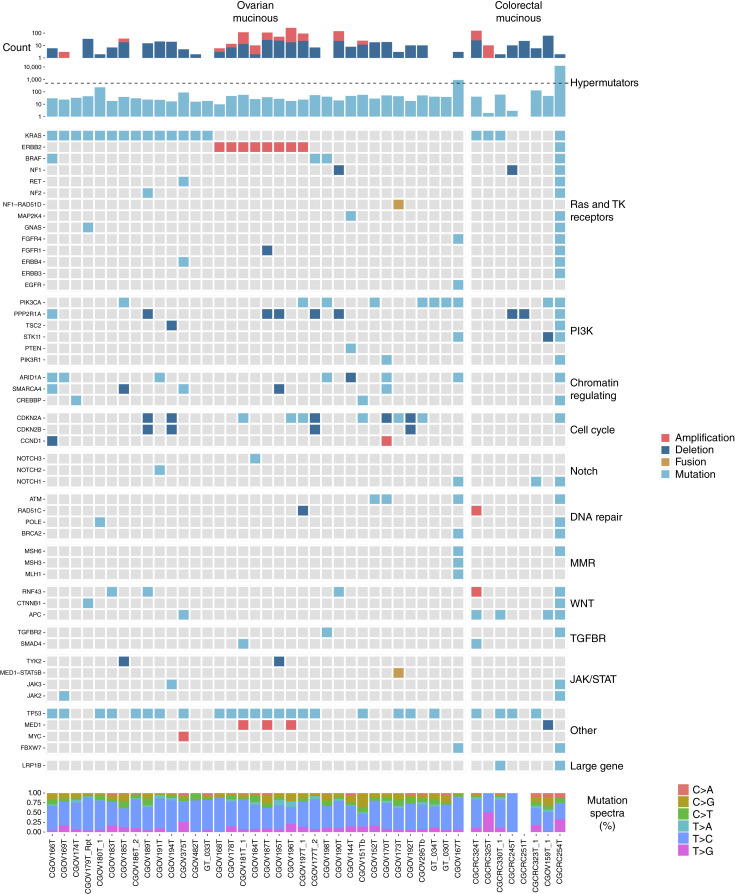
Integrated genomic analyses of ovarian and colorectal mucinous cancers. Integrated analyses of somatic point mutations, structural variants, including linked amplicons, deletions, and rearrangements, organized by pathways. The top of the figure shows the number of each type of alteration, the middle portion reflects the alterations present in each sample for each gene and pathway, and the bottom shows the mutation spectra.

We analyzed 36 patients with ovarian mucinous carcinoma and 10 patients with colorectal mucinous carcinomas for sequence (mutations and mutation signatures) and structural alterations (deletions, amplifications, rearrangements, and fusions). Using a threshold of 500 mutations per exome, we identified two hypermutated mucinous tumors. One was mucinous colorectal carcinoma CGCRC254T which had 12,666 exome mutations, likely a consequence of the R197S mutation in *POLE* (Fig. 4; Supplementary Tables S4 and S5). The hotspot somatic mutation observed in *POLE* occurred in the exonuclease domain of the encoded protein and has been reported in a small subset of microsatellite-stable and hypermutated colorectal carcinomas, affecting the proofreading activity of the enzyme during DNA replication (60). The other hypermutated mucinous carcinoma was ovarian tumor CGOV167T with 891 exome mutations. Using the Open Custom Ranked Analysis of Variants Toolkit, we determined that this tumor had at least two variants implicated in MMR deficiency, T981M in *MSH3* which is predicted to disrupt protein function according to SIFT, as well as a R248Tfs*8 in *MSH6* which is predicted as pathogenic/likely pathogenic by ClinVar (61–63).

Of the genes known to be altered through sequence changes in ovarian and colorectal mucinous carcinomas, we found alterations at similar frequencies in *KRAS*, *PIK3CA*, *RNF43*, and *TP53*. We identified several genes known to be mutated in ovarian mucinous carcinomas, including *ERBB2*, *ARID1A*, and *CREBBP* (27, 64–66). We also detected mutations in the chromatin modifier gene *SMARCA4* that had not been previously reported in ovarian mucinous carcinomas. With the exception of the *POLE* mutant tumor, these genes were infrequently altered in colorectal mucinous carcinomas.

Analyses of focal copy number changes revealed amplifications of *CCND1* and *ERBB2* and homozygous deletions of *CDKN2A/B* in mucinous ovarian carcinomas, a finding consistent with previous studies (37, 67–69). Amplification of *ERBB2* was observed in 8 of the 36 ovarian mucinous carcinomas and provides a potentially useful target for trastuzumab therapy (70). *CDKN2A* deletions were observed in five cases, and six additional tumors had a sequence alteration in this gene (68). In contrast, none of the colorectal mucinous carcinomas had copy number alternations in *ERBB2*, *CCND1*, or *CDKN2A/B* [0/7, 95% confidence interval (CI), 0.00–0.22].

Interestingly, homozygous deletions of *NF1* were present in a small number of both ovarian and colorectal mucinous carcinomas. In addition, we identified a novel *NF1–RAD51D* rearrangement resulting in the likely inactivation of *NF1* in one ovarian mucinous carcinoma (Fig. 4). Inactivating alterations of *NF1* have been shown to lead to RAS overactivity in HGSOC (2). Although the role of *RAD51D* is unclear in this context, germline *RAD51D* carriers have been reported to develop ovarian tumors at an early age (71). We also identified a novel *MED1–STAT5B* rearrangement in the same ovarian mucinous carcinoma (Supplementary Fig. S8). *MED1* alterations have been implicated in bladder and breast cancers and may be a potential target for treatment in endocrine-resistant breast cancer (72, 73).

### Genomic and epigenomic analyses

**Figure 5.**
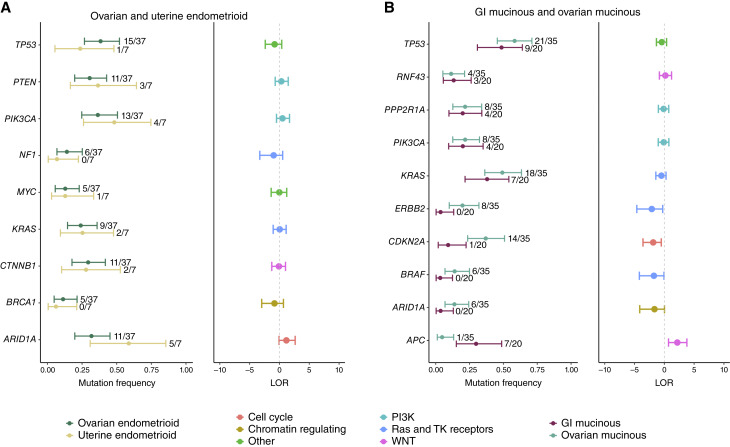
Comparison of mutation frequencies across histologic subtypes in commonly altered genes. **A,** For known cancer driver genes with an alteration frequency of at least 10% in patients with ovarian or uterine endometrioid cancer, we compared the proportion of samples with alterations between these histologic subtypes. Overall, mutation rates were comparable between ovarian and uterine endometrioid subtypes with 90% CIs of the LOR including zero (no difference). By contrast, we observed more differences between GI mucinous and ovarian mucinous tumors despite only six driver genes having an overall mutation frequency of at least 10%. **B,** Higher mutation rates were observed in ovarian mucinous tumors at *CDKN2A*, *KRAS*, *ERBB2*, and *TP53*. For all panels, horizontal line segments indicate 90% CIs and filled circles denote posterior medians. Hypermutators were excluded from these analyses.

We assessed whether cancers with the same morphologic tumor type had differences in the genetic and epigenetic alterations that commonly arise in the development of these cancers. To facilitate comparisons of the molecular alterations among the different tumor types, we integrated the sequence and structural changes at the gene level and limited our analyses to known cancer genes with an overall mutation rate of at least 10% within endometrioid and mucinous histotypes. For ovarian and uterine endometrioid carcinomas, somatic mutation frequencies were similar at nine commonly mutated genes in these tissues, namely *TP53*, *PTEN*, *PIK3CA*, *NF1*, *MYC*, *KRAS*, *CTNNB1*, *BRCA1*, and *ARID1A* (Fig. 5A). Despite a similar somatic mutation burden and tumor purity among mucinous cancers (Supplementary Fig. S1), higher mutation frequencies were observed in ovarian mucinous compared with GI mucinous carcinomas in four of the six genes frequently mutated in these tumors, including *CDKN2A* [posterior mean LOR ($\bar{\text{LOR}}$ = 1.87; 90% CI, 0.51–3.58)], *TP53* ($\bar{\text{LOR}}$ = 0.42; 90% CI, −0.38 to 1.33), *KRAS* ($\bar{\text{LOR}}$ = 0.49; 90% CI, −0.28 to 1.43), and *ERBB2* ($\bar{\text{LOR}}$ = 2.11; 90% CI, 0.27–4.62). As four of the six genes evaluated had a statistically significant difference in mutation rates (at type I error of 10%), these analyses suggest that ovarian and GI mucinous carcinomas, although histologically similar, may have different evolutionary cells of origin or selective pressures (*P* = 0.007; Fig. 5B). By contrast, the lack of differences among all nine genes evaluated in the comparison of ovarian to uterine endometrioid tumors suggested that these tumors are likely to have a similar evolutionary origin (*P* < 0.01).

Given the mutational differences observed, we hypothesized that ovarian mucinous carcinomas may be epigenetically distinct from GI mucinous carcinomas whereas ovarian endometrioid carcinomas may be similar to their uterine morphologic counterpart. We performed genome-wide methylation analyses of the GI mucinous, ovarian mucinous, and ovarian endometrioid carcinomas using arrays that interrogated more than 850,000 CpG sites within gene promoter regions, gene bodies, and intragenic and various annotated regulatory elements across the genome (74). After normalization, we estimated the proportion of methylated CpG sites as the fraction of CpG probes with β > 0.3. We observed modest differences in the overall proportion of methylated CpG sites between mucinous and endometrioid tissues (F_3,89_ = 2.9; *P* = 0.03; Supplementary Fig. S9).

**Figure 6.**
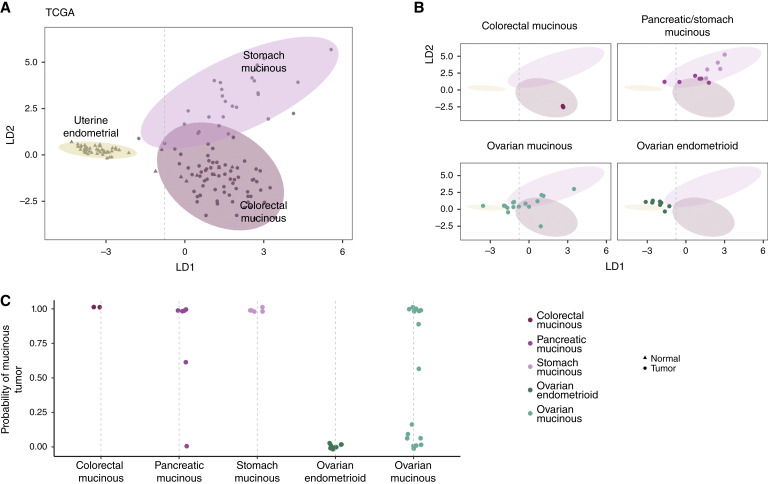
Ovarian endometrioid carcinomas are epigenetically similar to healthy uterine endometrium, whereas ovarian mucinous carcinomas are mixed with both mucinous- and endometrial-like signatures. **A,** We trained a LD model for the classification of samples as stomach mucinous, colorectal mucinous, or uterine endometrial from variably methylated CpG sites in TCGA samples. Ellipses denote the 95th percentile of the bivariate normal distribution for each of these histology classifications. **B,** Using the LD model from (**A**), the histologic classifications of samples were obtained and overlaid on the ellipses redrawn from **A**. The co-clustering of colorectal and stomach mucinous carcinomas with the corresponding tumor types from TCGA confirm that the methylation patterns that separate these tissues in TCGA broadly reflect histology differences (top). Whereas ovarian mucinous and ovarian endometrioid samples have no counterpart in TCGA (bottom), we evaluated whether these samples would be epigenetically similar to their morphologic counterparts in TCGA. **C,** Class probabilities from the LD model evaluated on the analyzed samples. As expected, patients with stomach and colorectal mucinous carcinomas were correctly classified as mucinous, and patients with ovarian endometrioid carcinomas were correctly classified as endometrial. Interestingly, classification of patients with ovarian mucinous carcinoma was more heterogenous, with nearly half of the samples exhibiting a mucinous signature and the remaining samples bearing more of a resemblance to endometrial methylation patterns. The two colorectal mucinous samples in **B** (top left) were sequencing replicates from a single patient’s tumor (patient CGCRC330).

To evaluate the methylation profiles of these samples in the context of tumor and normal samples from TCGA, we included 150 samples (46 normal uterine endometrial tissue, 23 stomach mucinous tumors, four pancreas mucinous tumors, and 77 colorectal mucinous cancers) that had been previously analyzed using methylation arrays (Supplementary Table S3). As the methylation probes would have substantial colinearity in a model for classifying tissue type on the basis of methylation patterns (Supplementary Fig. S10), we performed a LD analysis using the first five principal components as features. The first LD (LD_1_) captures a gradient in the composition of normal, endometrial histology to mucinous histologies of tumor origin, whereas the second LD (LD_2_) separates stomach and colorectal mucinous tumors (Fig. 6A). We projected the methylation profiles of these samples onto the principal components from TCGA to examine how these samples compared with methylation profiles of uterine endometrioid or mucinous carcinomas represented in TCGA (Fig. 6B). Overlaying the study colorectal cancer and stomach mucinous carcinoma profiles onto the 95% CIs for the TCGA tumor type classifications confirmed biological differences in these tumor types. Next, we assessed the ovarian mucinous and endometrioid carcinomas for which no corresponding tumor types were available in TCGA. We observed that ovarian endometrioid carcinomas clustered with TCGA uterine endometrial samples, corroborating their likely shared evolutionary origin. Ovarian mucinous carcinomas were more heterogeneous in their histologic classification, mapping to confidence groups across the different TCGA histologies (Fig. 6C). Overall, these analyses provide further evidence that ovarian endometrioid carcinomas share an evolutionary origin with uterine endometrioid carcinomas and that ovarian and GI mucinous carcinomas are more heterogeneous and unlikely to be evolutionarily related.

### Survival analyses

We integrated sequence and structural alterations within pathways to assess differences in overall survival for 49 patients. Genomic alterations in the PI3K pathway were weakly associated with longer overall survival for patients with ovarian endometrioid carcinomas (${Χ}_{1}^{2}$ = 2.7, *P* = 0.101, Supplementary Fig. S11A), whereas no differences in overall survival were observed in patients with ovarian mucinous carcinomas (${Χ}_{1}^{2}$ = 2.5, *P* = 0.114, Supplementary Fig. S11B). Whereas 13 patients with ovarian endometrioid carcinomas had alterations in the tumor-suppressor gene *PTEN*, only one patient with ovarian mucinous carcinoma had an alteration in this gene. Previous studies have reported improved survival for patients with *PTEN* mutations (75, 76). Mutations in *CTNNB1*, a gene in the Wnt/β-catenin signaling pathway, tend to be mutually exclusive of *TP53*, a finding corroborated by this study (Fig. 2), and patients with *CTNNB1* mutations have been reported to have lower genomic complexity (21). We observed a weak association between *CTNNB1* mutation status and survival in individuals with ovarian endometrioid carcinomas (*P* = 0.05; Supplementary Fig. S11C).

## Discussion

These analyses provide an extensive genomic and epigenomic characterization of ovarian mucinous and endometrioid carcinomas. Through the integration of somatic mutations, structural variants, including deletions, linked amplicons, intra- and inter-chromosomal rearrangements, as well as methylation analyses, we were able to detect frequently altered genes and pathways that describe the genomic and epigenomic landscape of these tumor types. These efforts revealed that ovarian endometrioid and uterine endometrioid carcinomas were genetically and epigenetically similar, suggesting they may share a common cellular precursor. In contrast, at both genomic and epigenomic levels, ovarian and GI mucinous carcinomas had distinct characteristics, highlighting the unique origins of these cancers.

We identified alterations that have not been previously observed in these tumors. These included alterations in *RAD51C*, *NOTCH4*, *SMARCA4*, *SMARCA1* and *JAK1* in ovarian endometrioid carcinomas and *SMARCA4* genes in ovarian mucinous carcinomas. WGS analyses revealed rearrangements involving *PTEN*, *NF1*, and *NF2* in ovarian endometrioid carcinomas and *NF1* and *MED1* in ovarian mucinous carcinomas. Many of these alterations provide new avenues for potential therapeutic intervention or new insights into the biology of these cancers. For example, patients with germline *NF1* mutations have been shown to respond well to MEK1/2 inhibitors, providing a new treatment avenue for tumors with alterations in this gene (77). *SMARCA4* loss, although not directly targetable, may result in cyclin D1 deficiency and susceptibility to CDK4/6 inhibition (78). *JAK1* is essential for IL-6-class inflammatory cytokine signaling and plays a critical role in metastatic cancer progression in breast cancer and may provide a similar role in ovarian endometrioid carcinoma (79). *MED1* is a tissue-specific co-activator of the estrogen receptor that mediates breast cancer metastasis and treatment resistance (80) and when targeted in model systems, results in reduced growth and metastasis of breast cancer xenografts (54). *MED1* rearrangements may lead to overexpression of this protein that could be exploited in patients with ovarian mucinous carcinoma.

In uterine endometrioid carcinomas, we identified *ESR1* activating mutations in two patients at amino acid residue 537 resulting in a mutation hotspot commonly seen in patients with breast cancer who have become resistant to estrogen-targeted therapies. Although aromatase inhibitors have been shown to select for Tyr537 *ESR1* mutations (43, 44), none of the patients in this study had been treated with these therapies. We hypothesize that these two women, each over the age of 60, may have been in a postmenopausal estrogen-low environment similar to antiestrogen therapy that increased selection for *ESR1* mutations. The significant interest in overcoming *ESR1* mutations for patients with resistance to aromatase inhibitors may ultimately provide new avenues for patients with these *de novo* alterations in uterine endometrioid carcinomas.

Additionally, we observed an association between alterations in specific pathways known to be important in other cancers with patient survival. Alterations in the PI3K and CTNNB1 pathways among patients with ovarian endometrioid carcinomas were associated with prolonged survival, whereas patients with ovarian mucinous carcinomas harboring alterations in the PI3K pathway had reduced survival and may benefit from additional targeted therapeutic approaches. Consistent with previous studies (21), *TP53* mutations among individuals with ovarian endometrioid carcinoma were associated with poor outcomes.

This study has several limitations. First, histopathologic diagnoses of endometrioid carcinomas were made without WT1 or TP53 protein levels from IHC, which have been shown to increase the accuracy of diagnosis (81). Similarly, IHC markers progesterone receptor and vimentin have been reported to improve classification accuracy of ovarian mucinous cancers but were not evaluated in this study (82). Because of these limitations, this study may include misdiagnosed cases. Similarly, Woodbeck and colleagues (82) report that as many of 20% of ovarian mucinous cancers may be misdiagnoses of ovarian endometrioid cancer (83). Finally, tumor grading that incorporates architectural features and rate of growth of ovarian and GI carcinomas were not available. Differences in grade between tumor subtypes could confound associations of molecular alterations with survival in these tumor types.

Despite these limitations, this study enables several important observations about endometrioid and mucinous carcinomas. Integrated analyses of the sequence and structural changes revealed that the genomic landscapes of ovarian and GI mucinous carcinomas were different but that ovarian endometrioid and uterine endometrioid carcinomas were related and may share similar origins. Global methylation patterns of ovarian and GI mucinous carcinomas indicate different cellular origins as has been recently suggested (27). GI mucinous carcinomas clustered with their respective matched normal tissue, suggesting that these tumors originate from their normal epithelium. On the other hand, genomic and methylation analyses suggest that ovarian and uterine endometrioid carcinomas share a similar cellular precursor, possibly explaining the high co-occurrence of these diseases. The accumulating evidence that other ovarian cancers such as HGSOC arise in the fallopian tube (3), together with pathologic evidence, suggests that the endometrium, through retrograde menstruation and endometriosis, is a likely source of both ovarian endometrioid and uterine endometrioid carcinomas. Taken together, these observations have important clinical implications and may suggest new methods for disease management.

## Supplementary Material

Supplementary Figure S1 Relationship between tumor purity and number of somatic mutations. (a) Tumor purity for multiple histological tumor types were estimated using FACETS. Samples that FACETS did not process due to undetectable copy number changes are marked with an x. (b) Patients with uterine endometrioid adenocarcinomas were more than twice as likely to have a hypermutator defect compared to patients with other cancers, including patients with ovarian endometrioid cancer (95% CI: 1.1 - 4.2-fold increase).

Supplementary Figure S2 Mutations in ESR1 at Tyr537 and Leu536. ESR1 mutations identified in two patients with uterine endometrioid carcinomas occurred at Tyr537, a hotspot most commonly associated with aromatase inhibitor resistance in patients with breast cancer patients, and Leu536. This hotspot is in close proximity to the region of the estrogen receptor that is important for ligand-dependent transcriptional function. The Tyr537 mutations cause a conformational change that constitutively activates the receptor independent of estrogen receptor binding. The Tyr537 residue is highlighted in blue in a three-dimensional view of the ESR1 protein (bottom). Due to its physical proximity to the Tyr537 hotspot, the Leu536 mutation is likely to have the same activating effect on the estrogen receptor.

Supplementary Figure S3 Mutation signature analyses. (a) Mutation signatures for ovarian and uterine endometrioid carcinomas visualized with unsupervised clustering. (b) Mutation signatures for ovarian and GI mucinous carcinomas. The intensity of the heatmap colors indicates the percentage of the overall mutational profile for a sample that is explained by the mutation signature.

Supplementary Figure S4 Circos plots of ovarian endometrioid carcinoma samples. Circos plots depict copy number alterations (black line segments interior of the chromosomes) as well as intra- and inter-chromosomal rearrangements (blue lines that connect different portions of the cancer genome).

Supplementary Figure S5 Circos plots of uterine endometrioid carcinoma samples.

Supplementary Figure S6 Circos plots of ovarian mucinous carcinoma samples.

Supplementary Figure S7 Circos plots of colorectal mucinous carcinoma samples.

Supplementary Figure S8 Rearrangements of ovarian endometrioid and ovarian mucinous carcinomas identified by TRELLIS. Rearrangements present in ovarian endometrioid carcinomas CGOV161T and CGOV172T (a, b) and an ovarian mucinous tumor CGOV173T (c). Split reads that span the fusion junction are shown in black, while read pairs that reside on either side of the junction are shown in green and blue.

Supplementary Figure S9 Proportion of methylated CpG sites in ovarian and mucinous carcinomas. The proportion of methylated CpG sites (mean Beta-values >0.3) are shown for patients with mucinous stomach, mucinous pancreatic, and ovarian mucinous and ovarian endometrioid carcinomas. Methylation was only available for one individual with colorectal mucinous carcinoma (not shown).

Supplementary Figure S10 Heatmap of methylation values in mucinous and endometrial histotypes. (a) Methylation levels (Betas) at 945 CpG sites having the highest variance across 164 TCGA patient samples that included 77 colorectal mucinous tumors, 37 stomach mucinous tumors, and 46 uterine endometrial samples from normal tissue. (b) Methylation levels at the same CpG sites were obtained from 16 patients with ovarian mucinous carcinomas, 8 patients with ovarian endometrioid carcinomas, 5 patients with stomach mucinous carcinomas, 6 patients with pancreatic mucinous carcinomas, and 1 patient with colorectal mucinous carcinoma.

Supplementary Figure S11 Kaplan-Meier survival curves for ovarian cancer patients with and without mutations in the PI3K and WNT pathways. (a, b) Alterations in the PI3K pathway trended towards increased survival among patients with ovarian endometrioid carcinomas and decreased survival among patients with ovarian mucinous carcinomas. (c) CTNNB1 alternations in the WNT pathway trended towards association with increased survival among patients with ovarian endometrioid carcinomas.

Supplementary Table S1 Summary of ovarian, uterine, and GI tumors analyzed through whole exome and whole genome analyses.

Supplementary Table S2 Summary of next-generation sequencing analyses.

Supplementary Table S3 TCGA samples included in methylation analysis.

Supplementary Table S4 Somatic sequence alterations from whole exome analyses.

Supplementary Table S5 Somatic sequence alterations from whole genome analyses.

Supplementary Table S6 Amplifications from whole genome analyses.

Supplementary Table S7 Copy number segments from whole exome analyses.

Supplementary Table S8 Deletions from whole genome analyses.

Supplementary Table S9 Gene fusions identified from whole genome analyses.

## Data Availability

Sequence data for WGS and WES can be found in the European Genome-phenome Archive (EGA) under study accession EGAS50000000523, and methylation array data can be found in the European Nucleotide Archive under study accession EGAS00001008175. Other data presented here are available by request to the corresponding authors.
