# Supplementary figures and images for: Genomic Landscapes of Endometrioid and Mucinous Ovarian Cancers and Morphologically Similar Tumor Types
Source: Cancer Res Commun. 2025 Nov 5;5(11):1952–66. doi: 10.1158/2767-9764.CRC-25-0147 (PMC12586982; doi:10.1158/2767-9764.CRC-25-0147)

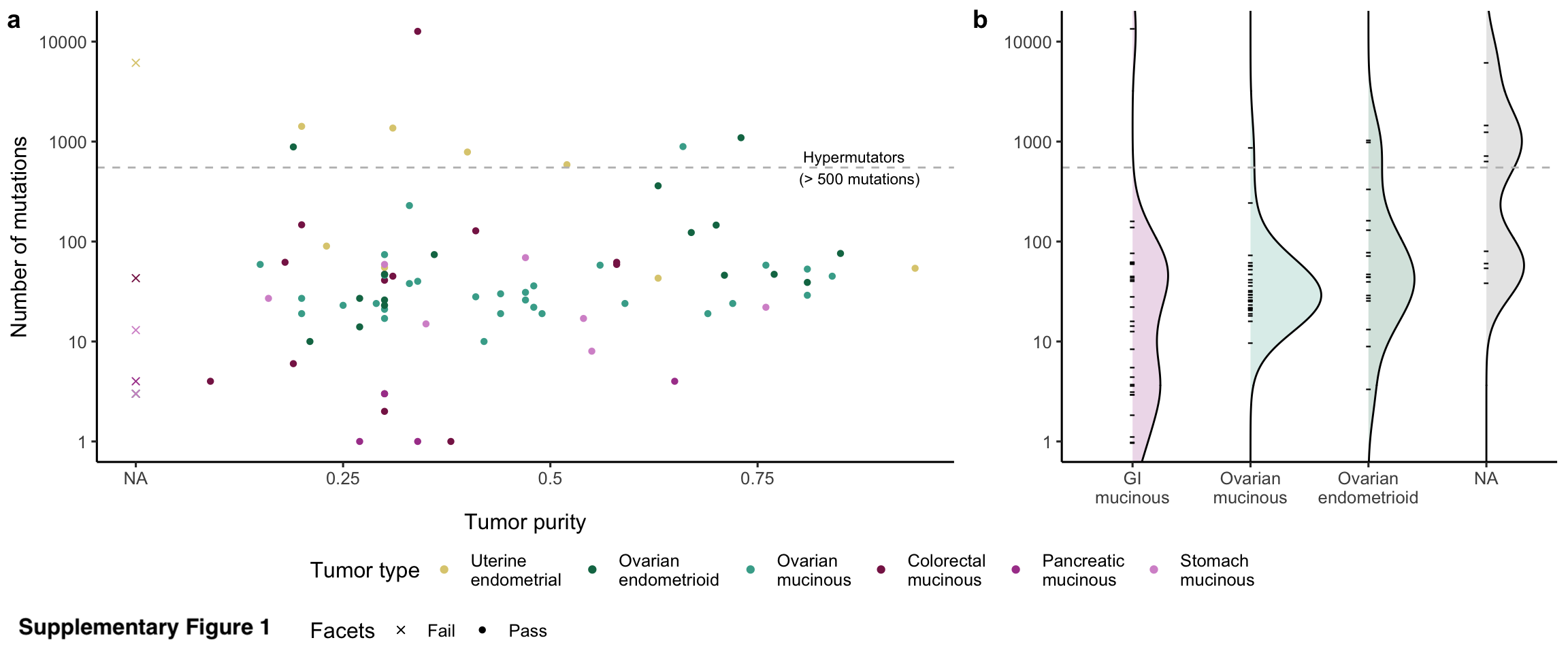

Supplement: Supplementary Figure S1 — Relationship between tumor purity and number of somatic mutations. (a) Tumor purity for multiple histological tumor types were estimated using FACETS. Samples that FACETS did not process due to undetectable copy number changes are marked with an x. (b) Patients with uterine endometrioid adenocarcinomas were more than twice as likely to have a hypermutator defect compared to patients with other cancers, including patients with ovarian endometrioid cancer (95% CI: 1.1 - 4.2-fold increase). [file crc-25-0147_supplementary_figure_s1_suppsf1.png]

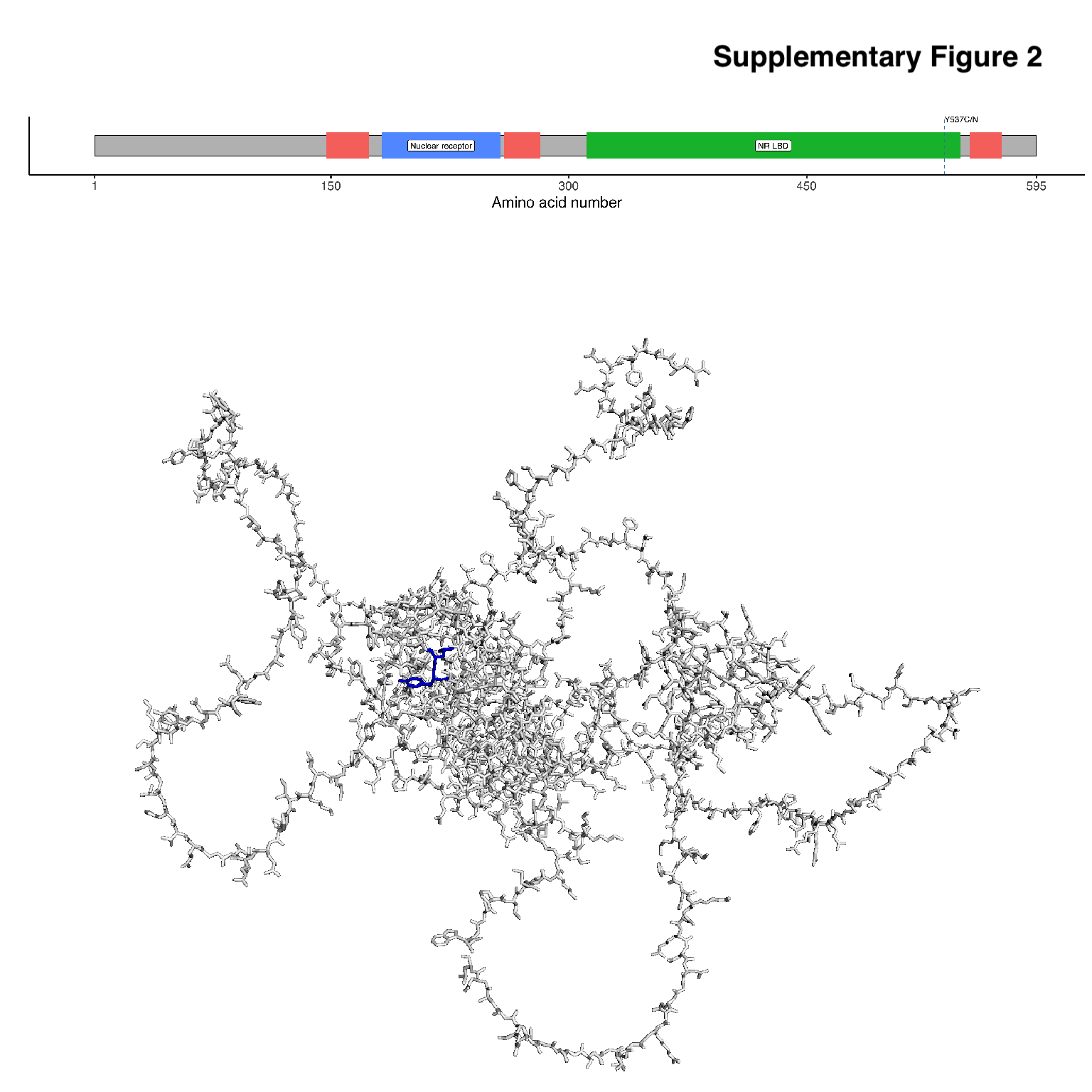

Supplement: Supplementary Figure S2 — Mutations in ESR1 at Tyr537 and Leu536. ESR1 mutations identified in two patients with uterine endometrioid carcinomas occurred at Tyr537, a hotspot most commonly associated with aromatase inhibitor resistance in patients with breast cancer patients, and Leu536. This hotspot is in close proximity to the region of the estrogen receptor that is important for ligand-dependent transcriptional function. The Tyr537 mutations cause a conformational change that constitutively activates the receptor independent of estrogen receptor binding. The Tyr537 residue is highlighted in blue in a three-dimensional view of the ESR1 protein (bottom). Due to its physical proximity to the Tyr537 hotspot, the Leu536 mutation is likely to have the same activating effect on the estrogen receptor. [file crc-25-0147_supplementary_figure_s2_suppsf2.png]

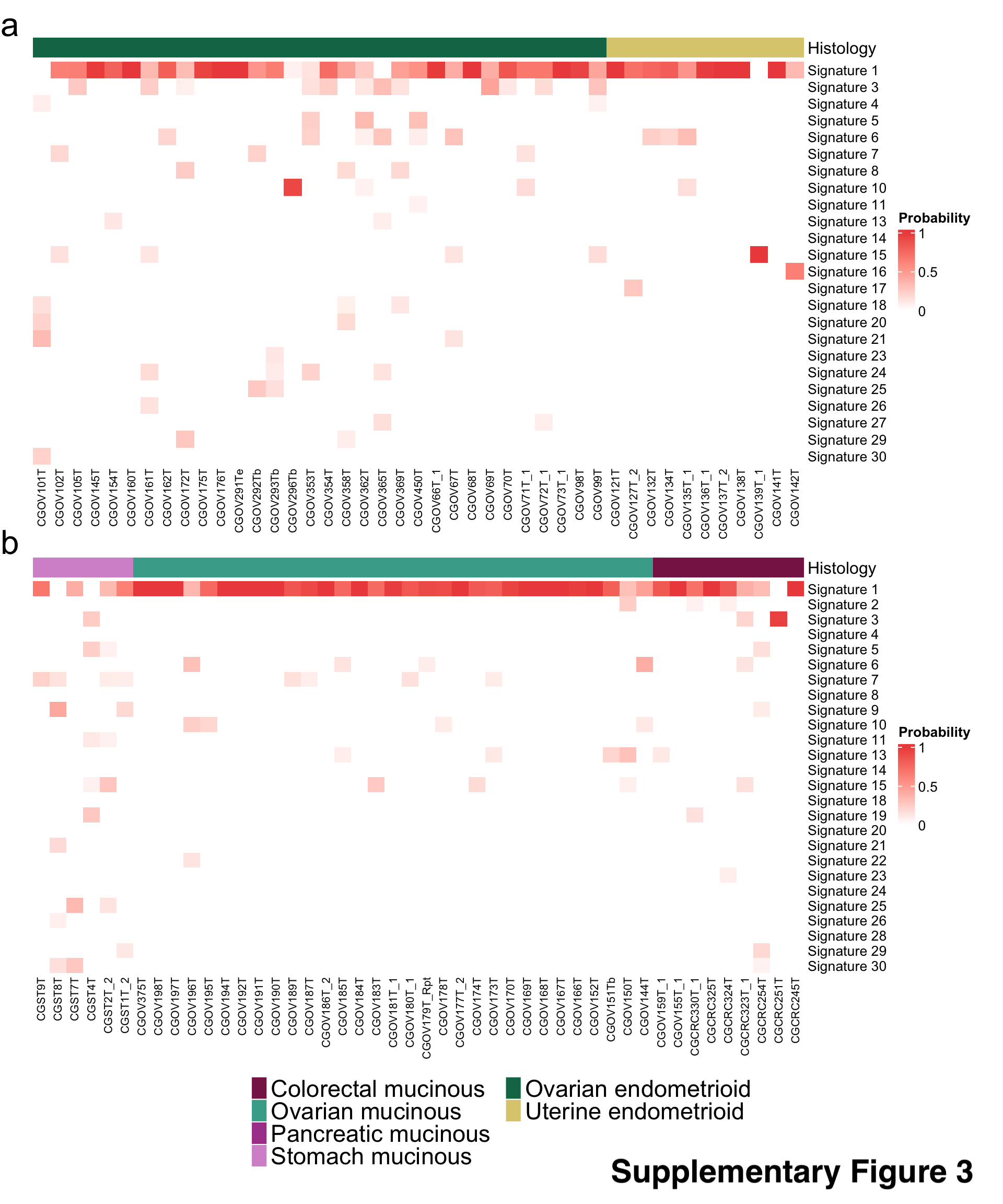

Supplement: Supplementary Figure S3 — Mutation signature analyses. (a) Mutation signatures for ovarian and uterine endometrioid carcinomas visualized with unsupervised clustering. (b) Mutation signatures for ovarian and GI mucinous carcinomas. The intensity of the heatmap colors indicates the percentage of the overall mutational profile for a sample that is explained by the mutation signature. [file crc-25-0147_supplementary_figure_s3_suppsf3.png]

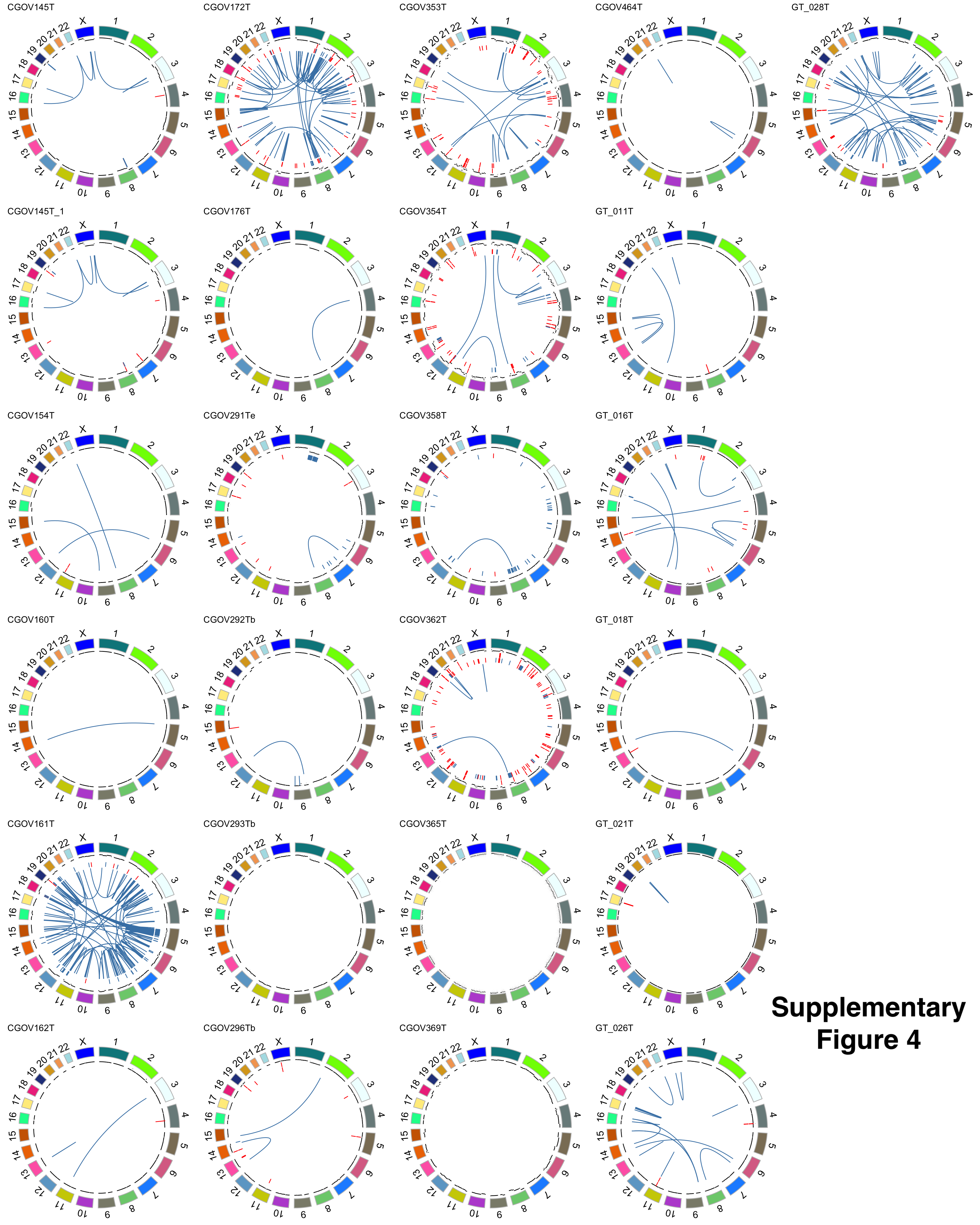

Supplement: Supplementary Figure S4 — Circos plots of ovarian endometrioid carcinoma samples. Circos plots depict copy number alterations (black line segments interior of the chromosomes) as well as intra- and inter-chromosomal rearrangements (blue lines that connect different portions of the cancer genome). [file crc-25-0147_supplementary_figure_s4_suppsf4.png]

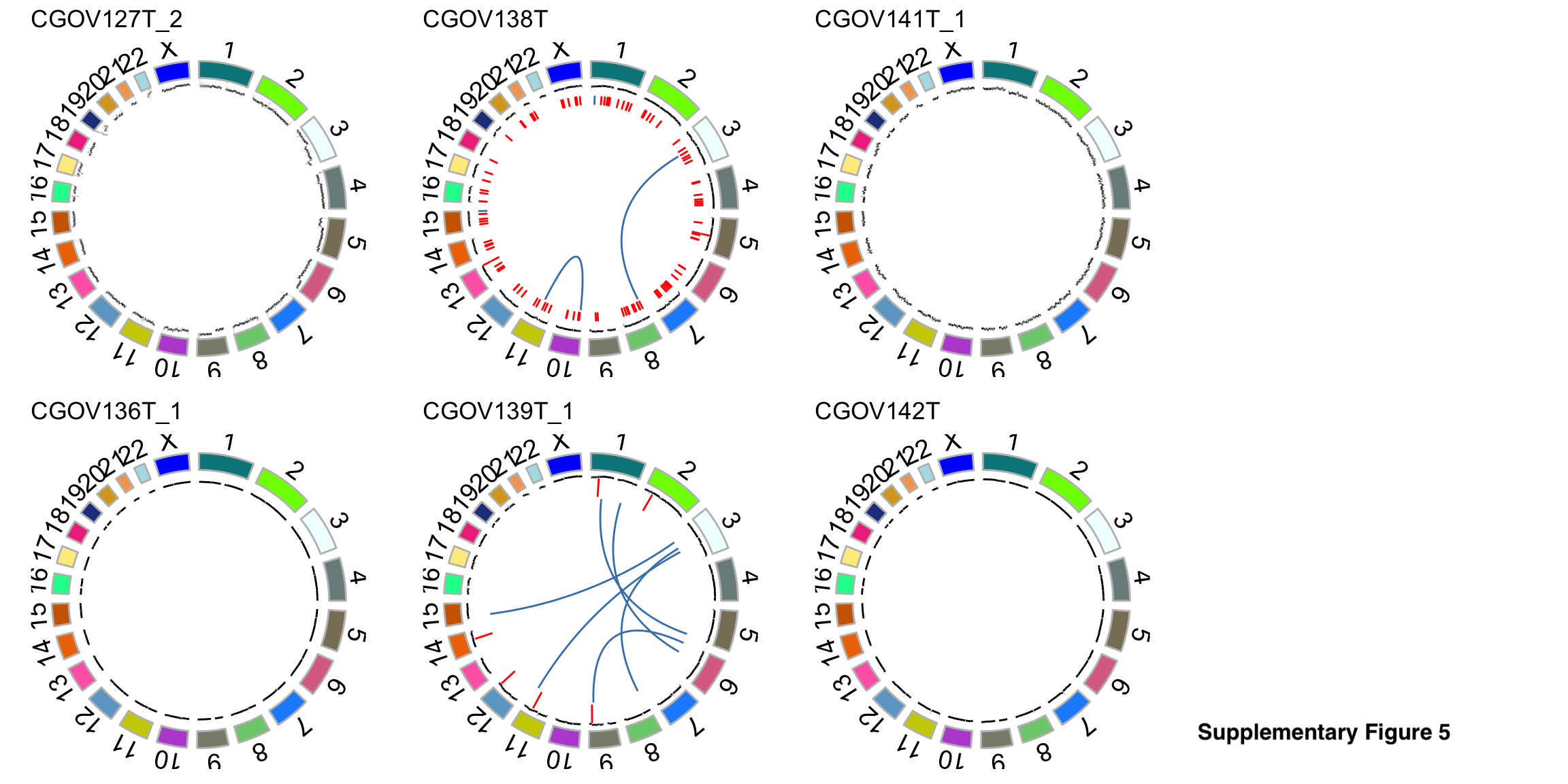

Supplement: Supplementary Figure S5 — Circos plots of uterine endometrioid carcinoma samples. [file crc-25-0147_supplementary_figure_s5_suppsf5.png]

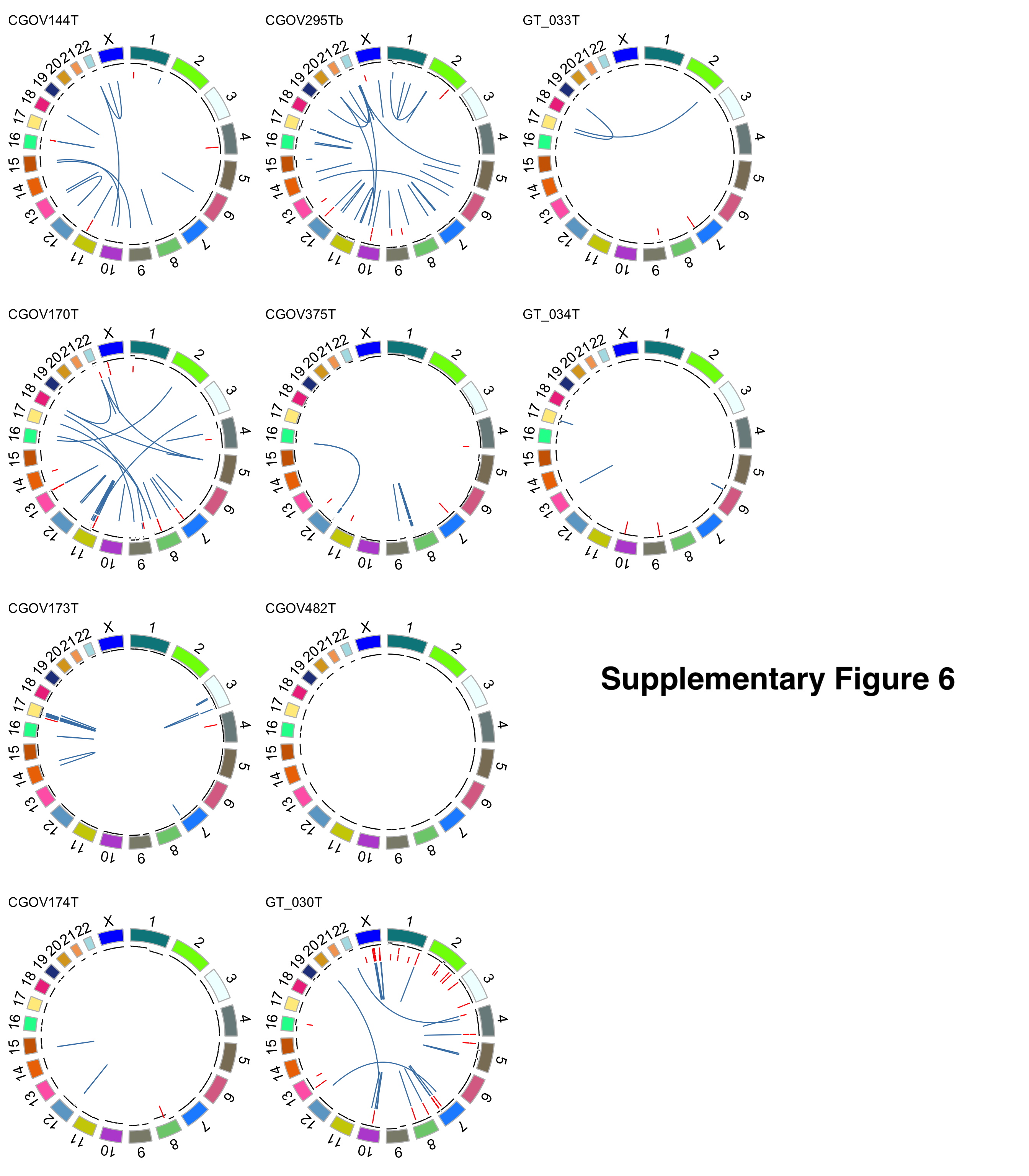

Supplement: Supplementary Figure S6 — Circos plots of ovarian mucinous carcinoma samples. [file crc-25-0147_supplementary_figure_s6_suppsf6.png]

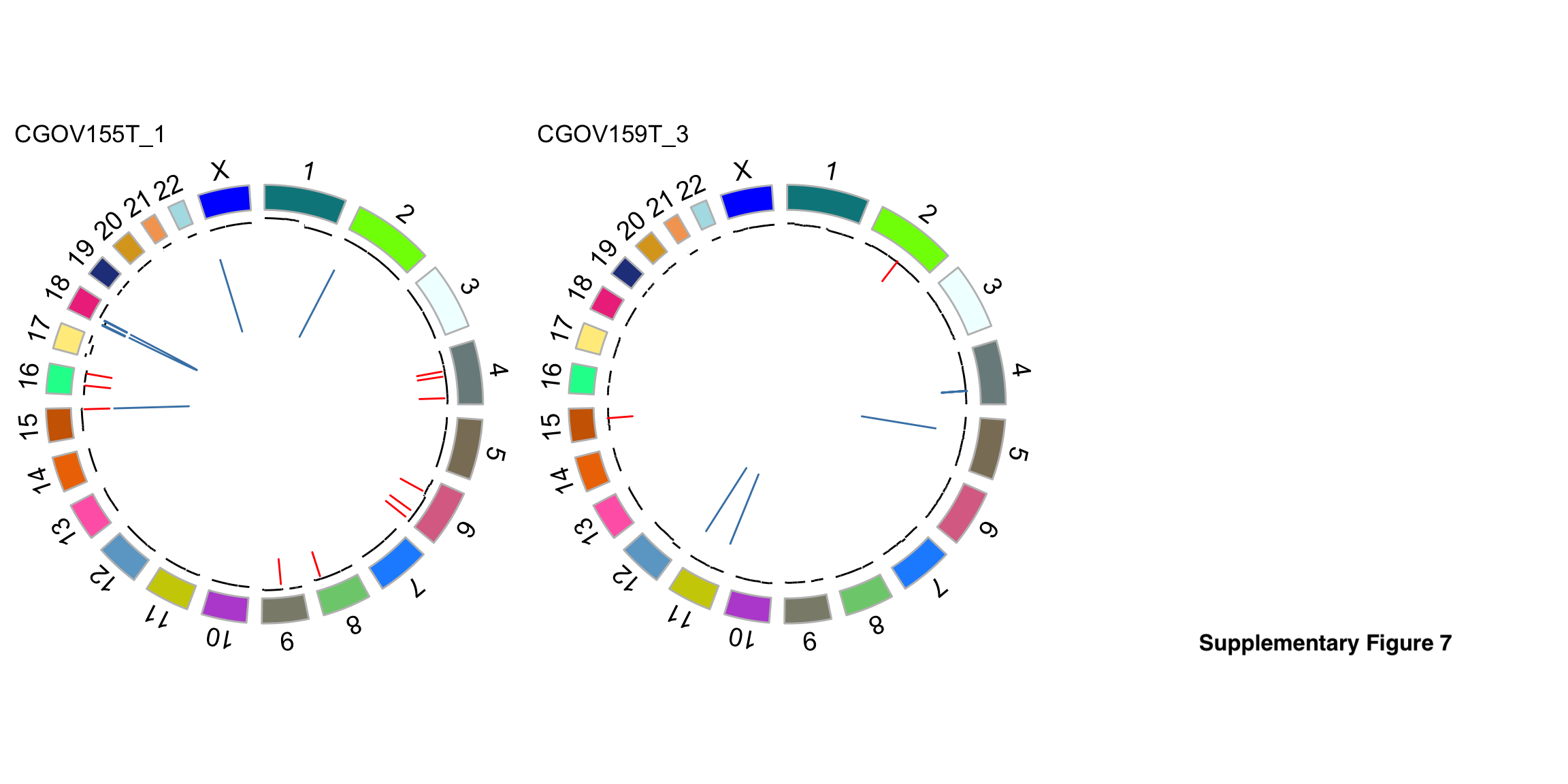

Supplement: Supplementary Figure S7 — Circos plots of colorectal mucinous carcinoma samples. [file crc-25-0147_supplementary_figure_s7_suppsf7.png]

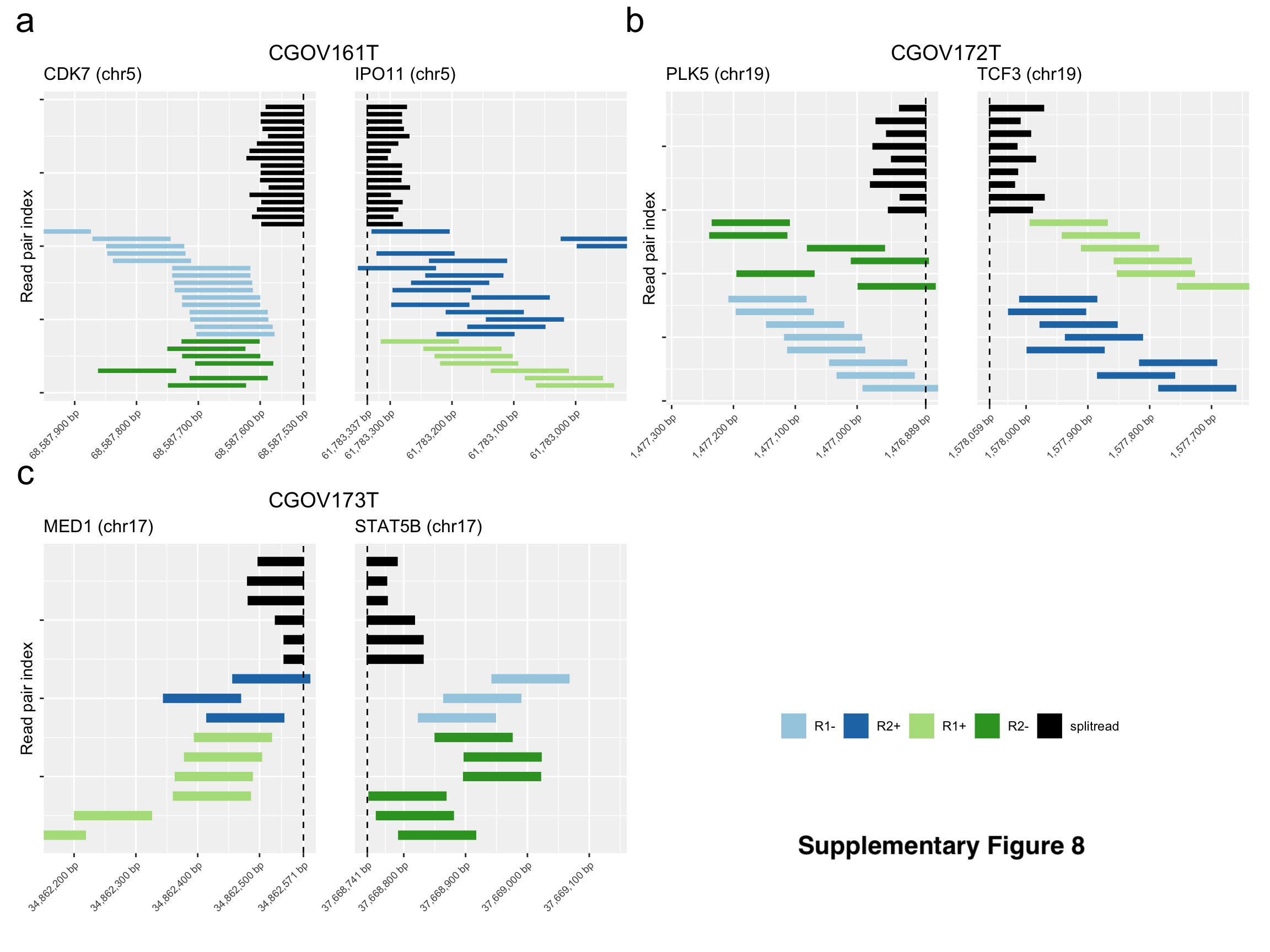

Supplement: Supplementary Figure S8 — Rearrangements of ovarian endometrioid and ovarian mucinous carcinomas identified by TRELLIS. Rearrangements present in ovarian endometrioid carcinomas CGOV161T and CGOV172T (a, b) and an ovarian mucinous tumor CGOV173T (c). Split reads that span the fusion junction are shown in black, while read pairs that reside on either side of the junction are shown in green and blue. [file crc-25-0147_supplementary_figure_s8_suppsf8.png]

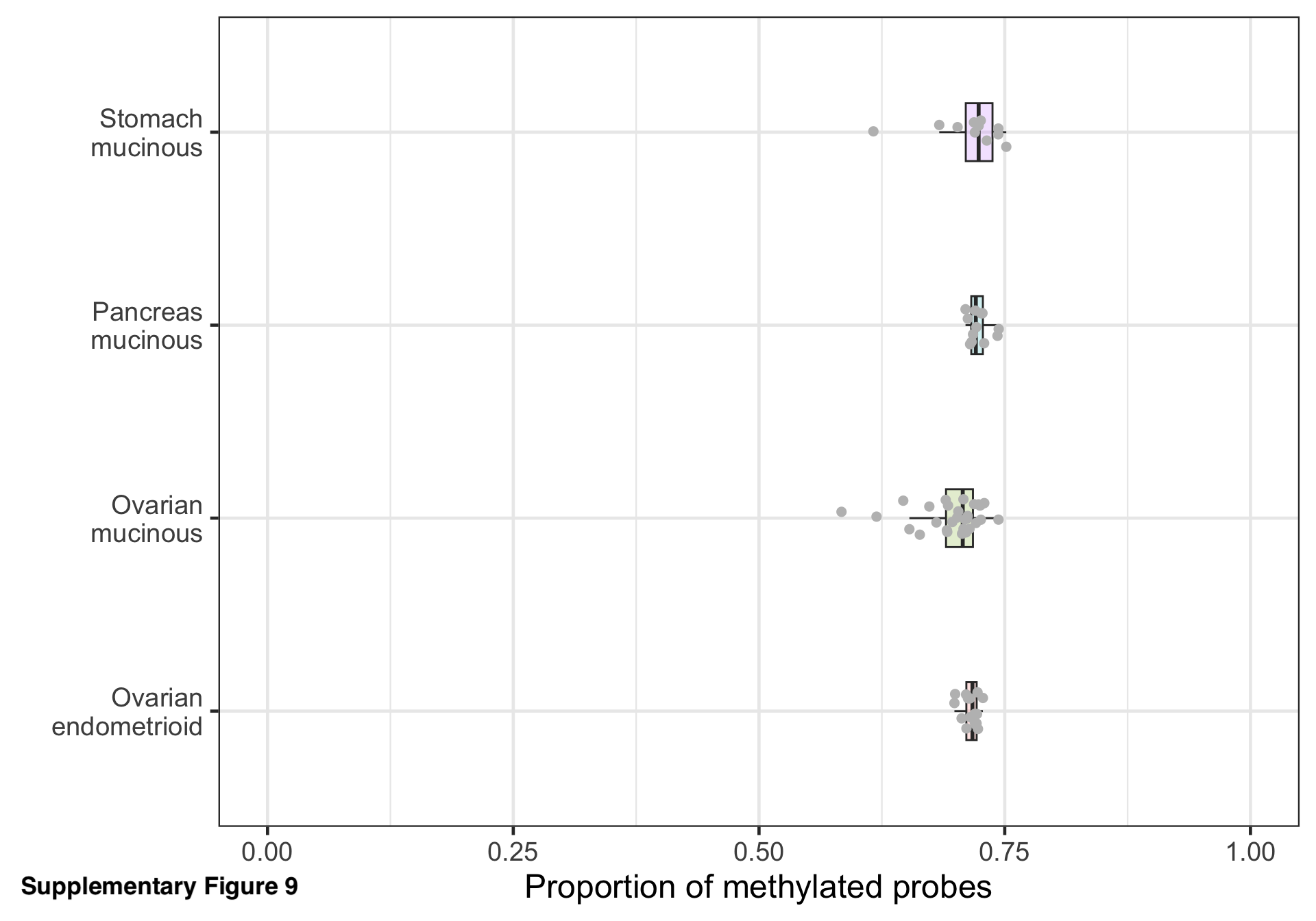

Supplement: Supplementary Figure S9 — Proportion of methylated CpG sites in ovarian and mucinous carcinomas. The proportion of methylated CpG sites (mean Beta-values >0.3) are shown for patients with mucinous stomach, mucinous pancreatic, and ovarian mucinous and ovarian endometrioid carcinomas. Methylation was only available for one individual with colorectal mucinous carcinoma (not shown). [file crc-25-0147_supplementary_figure_s9_suppsf9.png]

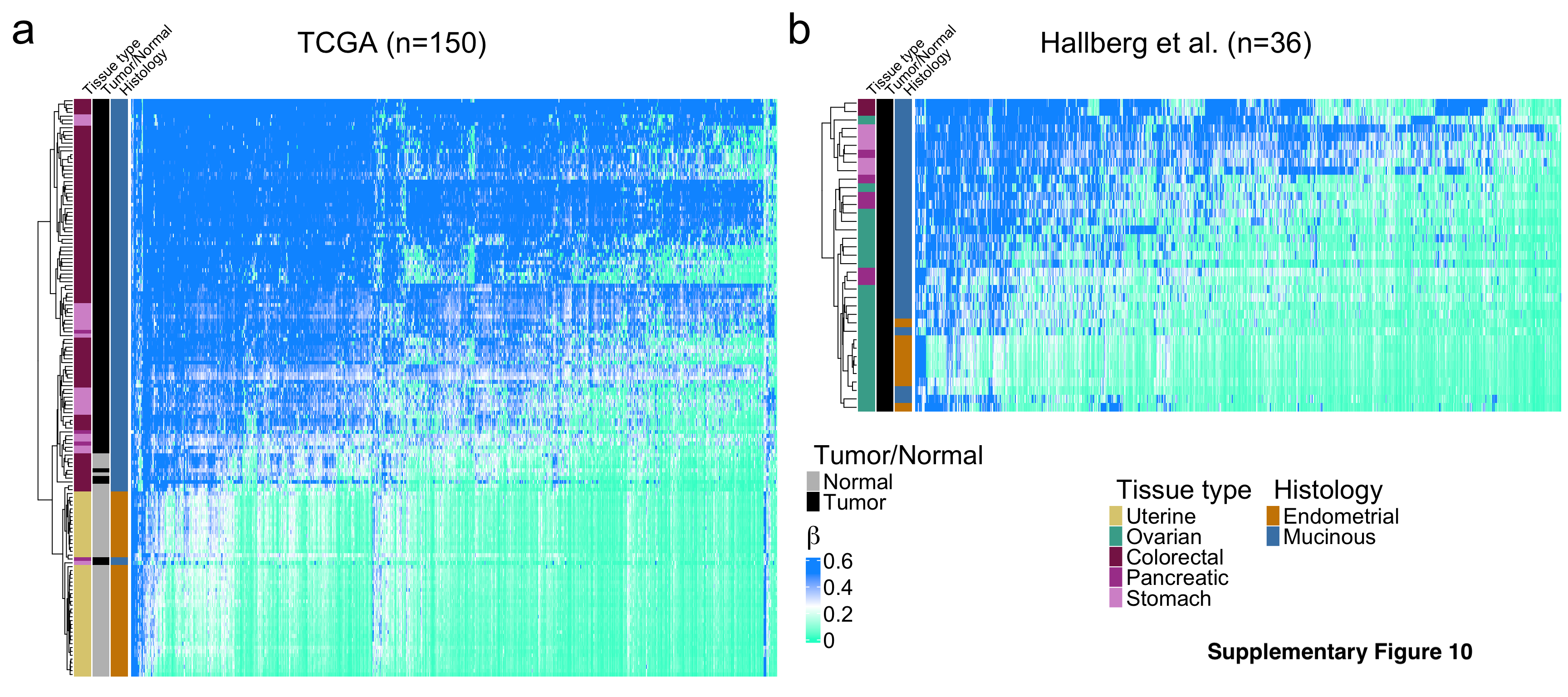

Supplement: Supplementary Figure S10 — Heatmap of methylation values in mucinous and endometrial histotypes. (a) Methylation levels (Betas) at 945 CpG sites having the highest variance across 164 TCGA patient samples that included 77 colorectal mucinous tumors, 37 stomach mucinous tumors, and 46 uterine endometrial samples from normal tissue. (b) Methylation levels at the same CpG sites were obtained from 16 patients with ovarian mucinous carcinomas, 8 patients with ovarian endometrioid carcinomas, 5 patients with stomach mucinous carcinomas, 6 patients with pancreatic mucinous carcinomas, and 1 patient with colorectal mucinous carcinoma. [file crc-25-0147_supplementary_figure_s10_suppsf10.png]

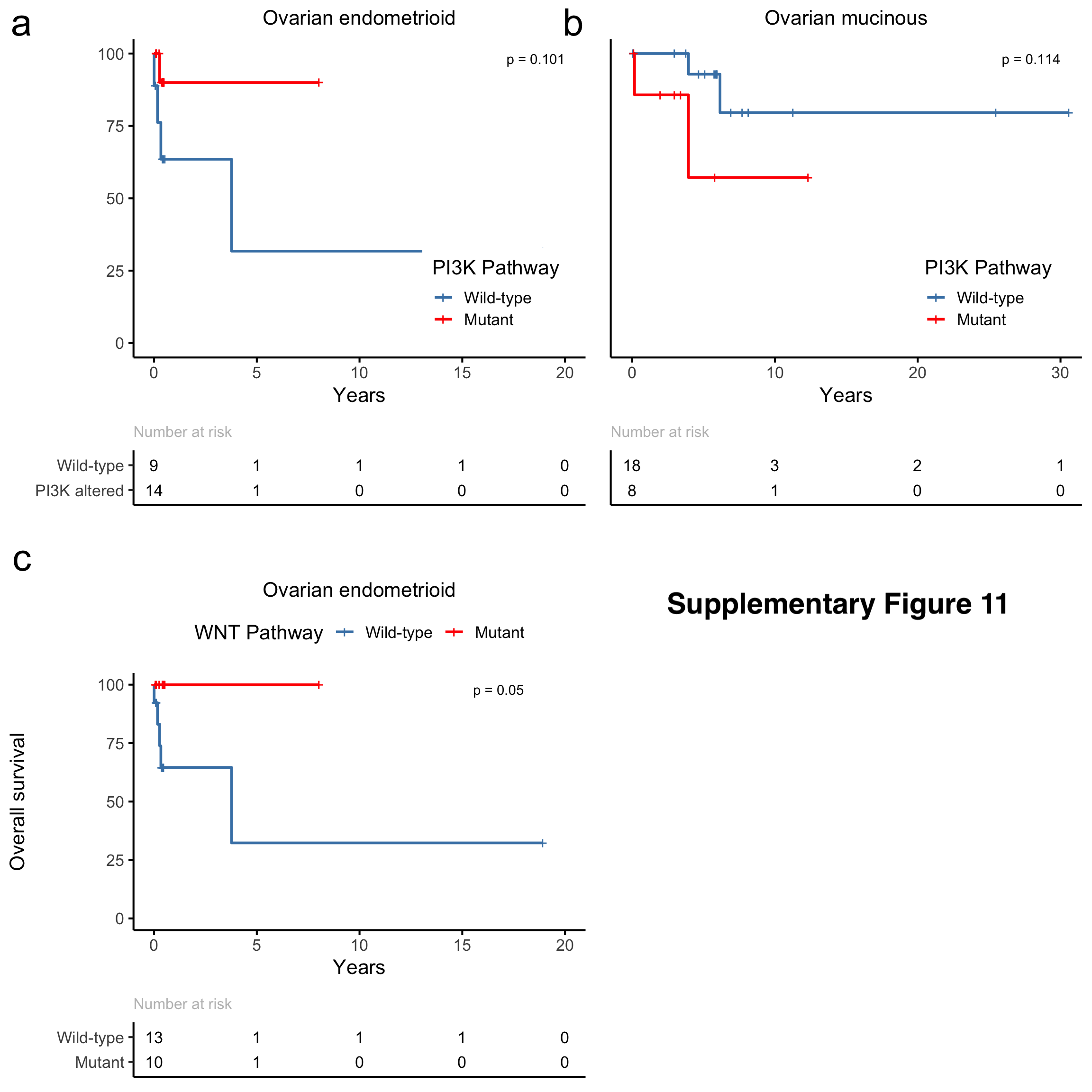

Supplement: Supplementary Figure S11 — Kaplan-Meier survival curves for ovarian cancer patients with and without mutations in the PI3K and WNT pathways. (a, b) Alterations in the PI3K pathway trended towards increased survival among patients with ovarian endometrioid carcinomas and decreased survival among patients with ovarian mucinous carcinomas. (c) CTNNB1 alternations in the WNT pathway trended towards association with increased survival among patients with ovarian endometrioid carcinomas. [file crc-25-0147_supplementary_figure_s11_suppsf11.png]
